# Exploring the Roles of Green Food Consumption and Social Trust in the Relationship between Perceived Consumer Effectiveness and Psychological Wellbeing

**DOI:** 10.3390/ijerph17134676

**Published:** 2020-06-29

**Authors:** Jianming Wang, Ninh Nguyen, Xiangzhi Bu

**Affiliations:** 1 School of Business Administration, Zhejiang University of Finance & Economics, Hangzhou 310018, China; sjwjm@zufe.edu.cn; 2 Department of Economics, Finance and Marketing, La Trobe Business School, La Trobe University, Melbourne 3086, Australia; ninh.nguyen@latrobe.edu.au or; 3 Business Sustainability Research Group, Thuongmai University, Hanoi 100000, Vietnam; 4 Department of Business Administration, Business School, Shantou University, Shantou 515063, China

**Keywords:** perceived consumer effectiveness, green food consumption, social trust theory, social ideal theory, psychological wellbeing, China

## Abstract

Green food consumption is a core issue that contributes to solving environmental pollution and achieving sustainable development. This study aims to investigate the mediating role of green food consumption and social trust in the relationship between perceived consumer effectiveness and psychological wellbeing to provide new insights into green food consumption, based on social ideal theory and social trust theory. Using a sample data of 514 consumers in China, the results of structural equation modeling showed that perceived consumer effectiveness was positively related to psychological wellbeing. Furthermore, green food consumption mediated the relationship between perceived consumer effectiveness and psychological wellbeing. In addition, social trust moderated the relationship between perceived consumer effectiveness and green food consumption. Social trust also moderated the indirect effect of perceived consumer effectiveness on psychological wellbeing through green food consumption. The findings of this study enrich the extant literature relating to green food consumption and have practical implications for business managers and policymakers.

## 1. Introduction

Global warming and climate change have generated a tremendous challenge to our society in the last decades [1]. The challenge of reducing environmental pollution and obtaining sustainable development is an important issue for governments, organizations, researchers, and practitioners [2]. Green purchases and green food consumption are core issues that contribute to such a challenge [3].

Various studies have examined the issue of green purchases and green food consumption in the current literature. For example, Mohd et al. [4] explored the factors and mechanisms that motivate consumers to engage in green consumption behavior. He et al. [5] determined the relationship between tourists’ perceptions and relational quality with their environmentally responsible behaviors. Li et al. [1] investigated gender inequality that affects a household’s decision to adopt green consumption. Zhang et al. [6] determined the impact of haze pollution on residents’ green consumption behavior. Rustam et al. [3] examined the relationship between corporate environmental sustainability disclosure and green consumption behavior with the moderating role of environmental awareness. Furthermore, several studies have determined socioeconomic factors (e.g., gender, age, family size, and income) that influence green food purchase behavior [1,4]. Prior studies have provided substantial evidence on the factors that influence green purchases and green food consumption. However, further research should explore additional factors and mechanisms that affect green food consumption and their impact on human physiological and psychological outcomes [3,4,7].

Social ideal theory believes that an individual expects an ideal society [8,9,10]. People who expect an ideal society often think and act in ways that generate benefits and bring goodwill to society [9]. For example, those people tend to engage in charitable donation and philanthropy behavior [10]. Furthermore, perceived consumer effectiveness reflects an individual’s belief about his or her ability to improve environmental and social problems [11]. This perception may affect consumers’ behavior toward green food consumption, which may influence their psychological outcomes [12,13]. For example, those who hold high perceptions of effectiveness tend to engage in recycling behavior and purchase environmentally friendly products [11,12]. Unfortunately, how perceived consumer effectiveness affects green food consumption and psychological outcomes has not been determined in the current literature. Therefore, this study investigates the relationship between perceived consumer effectiveness and psychological wellbeing with the mediating role of green food consumption, drawing upon the theoretical foundation of social ideal theory.

In addition, social trust theory states that people who hold a high level of social trust tend to be optimistic about the future and confident in society, and they tend to trust in others [14,15]. Consequently, social trust may play an important role in enhancing people’s perceptions of their ability to solve environmental and social problems. Social trust may also influence people’s behavior toward consuming green products [16,17]. Unfortunately, the role of social trust has been ignored in the current literature. Thus, this study also investigates the moderating role of social trust in the relationship between perceived consumer effectiveness and green food consumption. Moreover, this study examines the moderating role of social trust in the indirect effect of perceived consumer effectiveness on psychological wellbeing through green food consumption.

In sum, this study focusses on social ideal theory and social trust theory to investigate the mediating role of green food consumption and the moderating role of social trust in the relationship between perceived consumer effectiveness and psychological wellbeing. The objective is to provide new insights into the antecedent and consequence of green food consumption.

The structure of this paper is organized as follows. Section 2 reviews the current literature and develops hypotheses. Section 3 discusses the research design and sample procedure. Section 4 presents the empirical results. Section 5 and Section 6 provide discussions and implications.

## 2. Literature and Hypotheses

### 2.1. Perceived Consumer Effectiveness

Perceived consumer effectiveness is defined as an individual’s belief about his or her ability to improve environmental and social problems [11]. Nevertheless, perceived consumer effectiveness reflects an individual’s belief that their actions can make a difference in solving social and environmental pollution [18]. People who hold high perceptions of effectiveness tend to believe that they can influence and improve environmental and social problems. Hence, they tend to be highly confident and engage in socially responsible behavior [19]. For example, Roberts [20] suggested that green consumption is often associated with consumers’ perceptions of effectiveness. Webb et al. [21] and Cojuharenco et al. [22] reported that perceived consumer effectiveness is an important predictor of socially responsible behavior. Jaiswal and Kant [12] found that consumers who believe in their effectiveness tend to hold high intention to purchase green products. He and Zhan [23] demonstrated that perceived consumer effectiveness positively predicts consumers’ intention to adopt environmentally electric vehicles. Wang and Chen [24] found that perceived effectiveness of fair trade consumers tends to hold high intention to purchase fair trade products. Higueras-Castillo et al. [13] also reported that perceived consumer effectiveness is an important predictor of consumers’ attitudes toward electromobility. In sum, prior studies have provided substantial evidence to explain the predictive ability of perceived consumer effectiveness on socially responsible behavior.

### 2.2. Psychological Wellbeing

Wellbeing refers to a physical, mental, and psychological state in which individuals experience a sense of happiness, satisfaction, and pleasure [25,26]. Research in the last decades focused on psychological wellbeing. Three important scientific disciplines have been critical for the study of psychological wellbeing: developmental psychology often studies the psychological wellbeing of human life across different lifespans [25]. Personality psychology focuses on the relationship between self-actualization, individuation, and maturity with psychological wellbeing [27]. Clinical psychology emphasizes the association between mental illness and psychological wellbeing [28]. Moreover, several researchers have identified social life satisfaction as a core indicator of psychological wellbeing [29]. Social life satisfaction is the way in which individuals present their emotions and feelings and satisfaction with social relationships, goal achievement, self-concepts, and self-perceived ability to cope with an individual’s daily life [30]. Several studies have reported important factors that affect life satisfaction, including personality, self-esteem, age, experience, personal values, culture, family, and career [25,29,30,31]. In this study, social life satisfaction is treated as a key factor that reflects the psychological wellbeing of consumers [26,28,30].

### 2.3. Perceived Consumer Effectiveness and Psychological Wellbeing

Social ideal theory states that people tend to expect an ideal society in which they can enjoy justice, goodwill, and wellbeing [8,9,10]. Social ideal theory provides directions and guidelines to drive people’s attitudes and behavior toward positive activities that are beneficial to society [32]. People who hold high expectations about an ideal society often favor living in a harmonious and peaceful environment. They tend to engage in activities that create a high value for the environment and society [33]. Furthermore, those who hold the belief of an ideal society often show positive emotions and feelings about the social world. They also are highly confident, enjoyable, and optimistic about their lives because they are often motivated by hope and expectation about a better future [8].

From the logic of social ideal theory, consumers who hold high perceptions of their effectiveness often believe that they can change the social world. That is, they are optimistic about their capability to improve and solve environmental problems [10]. When consumers perceive their effectiveness to improve environmental and social problems, they may actively engage in activities that generate benefits for their society [12,23]. For example, consumers may believe that if they engage in recycling activities, purchase environmentally friendly products, and reduce their consumption of electricity and water, then they will contribute to solving environmental pollution [13]. Thus, these consumers tend to be satisfied with their social life because they may believe that they create good things for society. In other words, perceived consumer effectiveness enhances consumers’ positive emotions and wellbeing because they are optimistic and believe that they can contribute to the goodwill of their society. They have contributed to building an ideal society [9]. Thus, the following hypothesis is proposed.

**Hypothesis** **(H1):** *Perceived consumer effectiveness is positively related to psychological wellbeing*.

### 2.4. Mediating Role of Green Food Consumption

Green consumption is a broad concept in the current literature. Van Raaij and Verhallen [34] identified energy consumption behavior as the key activity of green consumption. Clark et al. [35] suggested that consumers engaging in a green electricity program are considered a green consumption behavior. Gilg et al. [36] defined green consumption as consumer purchasing products that have a less negative impact on the environment. Hartmann and Apaolaze-Ibanez [37] stated that consumers’ intention to purchase green energy brands could also be viewed as green consumption. Guo et al. [38] explained residential electricity consumption as a major type of green consumption based on the theory of planned behavior. In a broader concept, Mohd et al. [4] identified green consumption as an entire process of selection, use, and disposal of resources that generate impacts on the environment and society. Green food consumption is often viewed as a type of green consumption, which reflects consumers’ purchase behavior toward green food products that are perceived as healthy, safe, and environmentally friendly [39].

Social ideal theory can provide a solid theoretical foundation to explain the relationship between perceived consumer effectiveness, green food consumption, and psychological wellbeing. According to social ideal theory [8,9,10], consumers who hold high perceptions of effectiveness tend to engage in activities that create values and benefits the society because they believe that their behavior will contribute to building an ideal society [8]. With the idea of a better world, these consumers may invest time, energy, and effort to address environmental and social issues. For example, consumers may engage in donations to help other people. They also engage in green consumption behaviors, such as purchasing environmentally friendly products [12], using electric vehicles [23], consuming organic foods [39], and adopting electromobility to replace traditional polluted cars [13]. Furthermore, when consumers engage in green consumption behavior, they likely obtain additional benefits from such behavior. For example, the behavior of consumers purchasing and consuming organic and green foods contributes to their health because these types of foods provide additional nutrition and are safe to human health than traditional foods. Green food consumption also reduces the negative impact of farming and processing on the environment [39]. Consequently, when consumers perceive that their green food consumption contributes to the goodwill of human health, environment, and society, they tend to feel happy and satisfied with their behavior and lives [40]. Therefore, perceived consumer effectiveness will likely motivate consumers to engage in green food consumption, which in turn increases consumers’ positive emotions and wellbeing toward their lives. The following hypothesis is proposed.

**Hypothesis** **(H2):** *Green food consumption positively mediates the relationship between perceived consumer effectiveness and psychological wellbeing*.

### 2.5. Moderating Role of Social Trust

Social trust theory states the tendency that people hold a positive belief about the social world. These people tend to be optimistic and trust others in their society [14,15]. Social trust reflects an individual’s general belief that people will avoid behaving in ways that disadvantage others and take actions that benefit others [41]. Social trust may not be a stable trait, but it can be affected by several factors from an individual’s internal and external environment, such as social interaction and individual experiences [42]. Social trust is a broad concept that has not been defined in a consistent way [43]. However, this study followed Day and Settersten Jr.’s [16] suggestions and focused on several aspects of social trust, including confidence in society, optimism about the future, and general trust in others. Furthermore, social trust theory states that beliefs about society shape and influence a person’s view about the benevolence of other human beings [17]. People who hold different perceptions of social trust tend to think and act differently [16]. For example, people with low social trust are likely to be pessimistic, uncooperative, and egoistic. These people are less likely to help others [44]. By contrast, trusting people tend to be optimistic, cooperative, tolerant, and altruistic. They often engage in activities that bring benefits and positive results for others [45].

According to social trust theory, the influence of perceived consumer effectiveness on green food consumption may differ between people who trust and do not trust their society. Considering that people who hold a high level of social trust tend to be optimistic and altruistic, they may believe that they are useful, and their actions will bring benefits to the environment, society, and other people [46]. Hence, these people may actively engage in green food consumption because they believe that their green food consumption will create additional values for society [4,47]. For example, when consumers trust their society and other people, consumers may think that if they purchase environmentally friendly foods, then their purchase behavior will generate goodwill for the society. Consequently, consumers may actively engage in such consumption activities [8,12,23]. By contrast, people who hold a low level of social trust may be pessimistic and lack confidence. These people may believe that they are not able to contribute to society. Hence, the lack of trust people may behave in egoistic manners and even take actions that harm others and society [44,47]. In other words, when people hold a low level of social trust, they do not believe in their ability to improve social and environmental issues. They may not engage in green food consumption because they lack confidence and may perceive green food consumption as unnecessary activities [47]. Therefore, the influence of perceived consumer effectiveness on green food consumption will likely vary between people who hold a high level of social trust as compared with those who hold a low level of social trust. The following hypothesis is proposed.

**Hypothesis** **(H3):** *Social trust moderates the relationship between perceived consumer effectiveness and green food consumption such that the relationship is strong when social trust is high and vice versa*.

**Figure 1:**
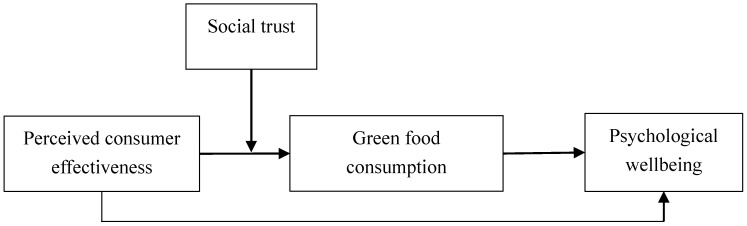
Conceptual framework of the study.

This study proposed a research model, as shown in Figure 1. As stated above, green food consumption is argued to have a mediating effect on the relationship between perceived consumer effectiveness and psychological wellbeing. Moreover, social trust is hypothesized to moderate the relationship between perceived consumer effectiveness and green food consumption. Thus, social trust will moderate the indirect effect of perceived consumer effectiveness on psychological wellbeing through green food consumption. The following hypothesis is proposed.

**Hypothesis** **(H4):** *Social trust moderates the indirect effect of perceived consumer effectiveness on psychological wellbeing through green food consumption such that the indirect effect is strong when social trust is high and weaker when social trust is low*.

## 3. Methods

### 3.1. Measures of Variables

This study adopted measurement items of variables in prior studies. Perceived consumer effectiveness was measured using four items from Straughan and Robert [48]. Green food consumption was measured using four items from Mohd et al. [4]. These constructs were measured using a five-point Likert scale from 1 (strongly disagree) to 5 (strongly agree). Moreover, social trust was measured using two items from Day and Setterstern Jr. [16]. This construct was measured using a five-point scale from 1 (never) to 5 (every day). Psychological wellbeing was measured using four items from Diener et al. [49]. This construct was measured using a five-point Likert scale from 1 (strongly disagree) to 5 (strongly agree). Table 1 shows the details of the items and variables.

### 3.2. Sample Procedure

This study used a questionnaire survey to collect data. We recruited professional translators to translate the measures. A forward and backward language translation was conducted to ensure the meaning of measurement items. Moreover, a pilot test was conducted at a large supermarket with the participation of 30 consumers. The purpose of this pilot test is to modify and ensure the clarity of the questionnaire. In the formal survey stage, we recruited a team to distribute questionnaires at different physical stores in Shanghai city. We randomly selected 10 physical stores to collect the sample data. The research team conveniently approached consumers and invited them to complete the questionnaire. We adopted a systematic sampling technique in which one of the three consumers was selected, considering that the list of consumers was not available. Consumers voluntarily participated in the survey. The survey was conducted from October 2019 to January 2020. Among the 800 questionnaires distributed, 520 were returned, and 514 were valid, with a response rate of 64.25%. Only six questionnaires were invalid and excluded from the final sample because of missing values. Kline [50] noted that the sample size should comply with N:q rule (N is the number item of the measurement scales, q is the number of cases). The ideal sample size should be 1:10 (1 measurement item requires 10 cases). In this study, the total measurement is was 14; hence, the number of sample sizes should be 140 cases. Thus, the sample size of 514 is considered adequate in this study.

### 3.3. Ethical Considerations

This study conducted a survey that involves human activity. Considering ethical standards, this study was conducted with the approval of the Major Project of The National Social Science Fund of China (20ZDA084). Consumers voluntarily participated in the survey and provided the measures with an anonymous questionnaire. Therefore, this study ensured the privacy and security of the respondents.

### 3.4. Analytical Methods

This study used SPSS statistical software (IBM, Armonk, NY, United States) to analyze the descriptive statistics and reliability of the measures. Furthermore, partial least square structural equation modeling (PLS-SEM) was adopted to perform the measurement model, which is used to test the validity of the measures. In addition, PLS-SEM was used to test the hypotheses in this study. Specifically, basic PLS-SEM was used to perform a confirmatory factor analysis (CFA). Based on the results of this CFA, the values of composite reliability (CR), the average variance extracted (AVE), and square roots of AVE were calculated to test the convergent and discriminant validity. Furthermore, PLS-SEM with the maximum likelihood estimation method was used to test all hypotheses in a single model. Structural equation modeling (SEM) is a combination of path and factor analyses. Several multiple regression analyses were combined into a single model so that the standard errors were controlled in SEM. PLS-SEM has been widely used in business and management studies [50].

## 4. Results

### 4.1. Sample Characteristics and Descriptive Statistics

Table 2 presents the basic information and characteristics of the respondents in this study: (1) most of the respondents were female (65.6%); (2) more than half of the respondents aged between 20 and under 30 (58.0%); (3) the majority of the respondents were not married (70.4%); (4) approximately 66.7% of the respondents had university education; (5) most of the respondents had monthly income between 500 and under 1000 USD (67.7%).

Table 3 shows the descriptive statistics of all variables. Results show that perceived consumer effectiveness was positively associated with green food consumption (r = 0.53, *p* < 0.01) and psychological wellbeing (r = 0.41, *p* < 0.01). Furthermore, green food consumption was positively associated with psychological wellbeing (r = 0.27, *p* < 0.01). In addition, social trust was positively associated with green food consumption (r = 0.51, *p* < 0.01) and psychological wellbeing (r = 0.47, *p* < 0.01).

### 4.2. Measurement Model

The results of the measurement model in this study indicate a good model fit of the hypothesized model. Specifically, all goodness of fit index of the measurement model met the required threshold: χ^2^/d.f. = 2.93 which was less than 3; Comparative Fit Index - CFI = 0.94, Goodness-of-fit Index-GFI = 0.91, and Tucker Lewis Index - TLI = 0.92 which were all greater than 0.90; and Root Mean Square Error of Approximation - RMSEA = 0.07 which was less than 0.08 [50].

This study used Cronbach’s alpha to measure reliability. Results in Table 4 show that Cronbach’s alpha of all variables ranged from 0.77 to 0.90, which exceeded the cutoff value of 0.60 [51]. Thus, the measures in this study show good reliability.

Convergent validity was tested using CR and AVE in this study. Hair et al. [51] suggested that the CR value must be greater than 0.70, and AVE values must be greater than 0.50. Results indicate that CR and AVE values of all variables were all greater than the threshold value. Thus, the measures in this study have good convergent reliability.

Discriminant validity was tested by comparing the square roots of AVE and Pearson correlations of all variables. Hair et al. [51] suggested that square roots of AVE must be greater than all correlation coefficients of all variables. Table 3 shows that the square roots of AVE were greater than all Pearson correlation, thereby providing evidence for the good discriminant validity of the measures in this study.

### 4.3. Common Method Bias

Following Podsakoff et al. [52], this study conducted Harman’s one-factor test to detect the problem of common method bias. Results of the unrotated solution of principle component analysis show that four factors emerged with 64.24% of the variance, and the first factor accounted for only 17.02% of the variance. Furthermore, results of one-factor model of CFA indicate a poor model fit (χ^2^/d.f. = 11.47, CFI = 0.76, GFI = 0.74, TLI = 0.734, and RMSEA = 0.14). Thus, the common method bias may not seriously affect the results of hypothesis testing in this study.

### 4.4. Structural Model

**Figure 2:**
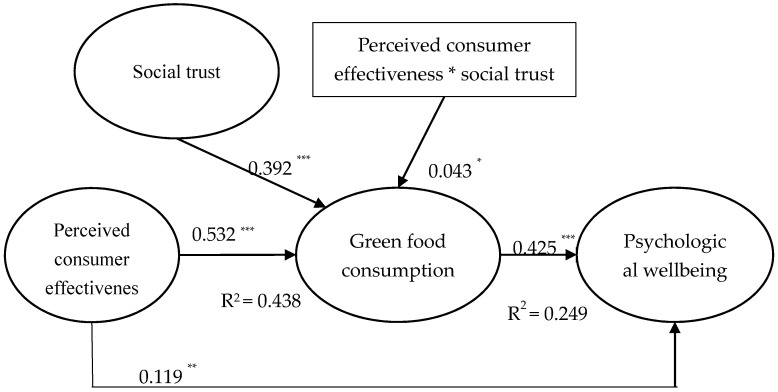
Results of hypothesis testing (*N* = 514). * *p* < 0.05, ** *p* < 0.01, *** *p* < 0.001.

**Figure 3:**
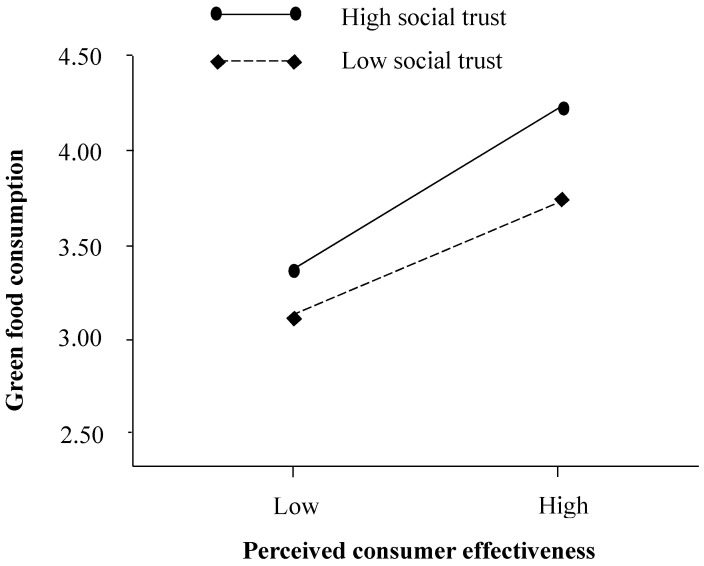
Moderating effect of social trust.

This study used PLS-SEM to test the hypotheses. Results in Figure 2 show that perceived consumer effectiveness was positively related to psychological wellbeing (β = 0.119, *p* <0.01), thereby supporting Hypothesis (H1). Furthermore, perceived consumer effectiveness was positively related to green food consumption (β = 0.532, *p* < 0.001) which in turn was positively related to psychological wellbeing (β = 0.425, *p* < 0.001). We followed Preacher et al. [53] to conduct a bootstrap analysis with 1000 bootstrap samples to confirm this indirect effect. Results indicate that the indirect effect of perceived consumer effectiveness on psychological wellbeing through green food consumption was statistically significant (perceived consumer effectiveness → green food consumption → psychological wellbeing: β = 0.328, *p* < 0.01, 95% CI = [0.252, 0.419]). Thus, Hypothesis (H2) was supported. In addition, results in Figure 2 show that the interaction effect between perceived consumer effectiveness and social trust was positively related to green food consumption (β = 0.043, *p* < 0.05). Results in Figure 3 also indicate that the influence of perceived consumer effectiveness on green food consumption was strong when social trust was high and vice versa. In other words, the influence of perceived consumer effectiveness on green food consumption varied among different levels of social trust. Thus, Hypothesis (H3) was supported.

This study followed Edward and Lambert’s [54] procedure to test the moderating effect of social trust on the indirect effect of perceived consumer effectiveness on psychological wellbeing through green food consumption. Table 5 shows that the indirect effect of perceived consumer effectiveness on psychological wellbeing through green food consumption varied among different groups of social trust (△β = 0.058, *p* < 0.01). Thus, Hypothesis (H4) was supported.

## 5. Discussion and Implications

This study aims to investigate the mediating role of green food consumption and social trust in the relationship between perceived consumer effectiveness and psychological wellbeing. The results provide important implications for researchers and managers.

### 5.1. Research Implications

First, this study argues for the direct influence of perceived consumer effectiveness on psychological wellbeing based on social ideal theory. Findings indicate that consumers who hold perceptions of effectiveness believe in their ability to solve social and environmental issues. Given this perception, they tend to feel satisfied with their social life because they may believe that they can contribute to the goodwill of their society [9]. The findings of this study provide new insights into the relationship between perceived consumer effectiveness and psychological wellbeing. To our best knowledge, this relationship has not been determined in prior literature. Thus, our study provides new knowledge and advances our understanding of the impact of perceived consumer effectiveness on psychological wellbeing.

Second, our findings demonstrate the mediating role of green food consumption in the relationship between perceived consumer effectiveness and psychological wellbeing. That is, according to social ideal theory, consumers who believe in their ability to change the social world tend to actively engage in green food consumption because they believe that their green food consumption is good behavior that contributes to building a better world [12]. Furthermore, green food consumption helps to protect the environment, solves social issues, and creates values for society [23,39]. Hence, when consumers contribute to bringing goodwill to society, they tend to feel satisfied with their social life [8]. Prior studies often determined the antecedents of green food consumption. However, this study extends social ideal theory and provides new evidence on the mediating mechanism of green food consumption into the relationship between perceived consumer effectiveness and psychological wellbeing. The findings of this study provide a theoretical foundation for researchers who may be interested in studying the role of social ideal theory in green food consumption.

Third, this study found the moderating role of social trust in the relationship between perceived consumer effectiveness and green food consumption. This finding indicates that consumers who trust other people in their society often feel optimistic and also tend to engage in altruistic activities [14,15]. These consumers may often believe in their ability to improve social and environmental problems. Consequently, they tend to engage in green food consumption behavior [4,47]. By contrast, consumers who hold a low level of social trust tend to be pessimistic and even engage in egoistic behavior. They are less likely to engage in green food consumption behavior due to the lack of confidence and beliefs in others [44,47]. Thus, the findings of this study provide new evidence on the moderating mechanism of social trust. This important effect of social trust has not been investigated in prior literature. Our findings help to extend social trust theory and clarify the role of social trust in green food consumption research.

Last, the research model in this study is unique and deals with the relationship among variables that have been greatly ignored in prior literature. The mediating mechanism of green food consumption behavior in the relationship between perceived consumer effectiveness and psychological wellbeing helps to advance our knowledge about the antecedent of green food consumption and the impact of green food consumption on consumers’ psychological wellbeing. To our best knowledge, none of the prior studies have determined the influence of green food consumption on consumers’ psychological wellbeing. Furthermore, the moderating mechanism of social trust is unique in our research model. Findings in this study clarify the role of social trust in enhancing consumers’ beliefs and behavior toward their ability to improve social and environmental problems. Thus, the mediating and moderating mechanisms in this study provide new insight into our knowledge and understanding of green food consumption behavior in the current literature.

### 5.2. Practical Implications

Based on empirical findings, this study provides several suggestions for business managers and policymakers. When consumers hold the belief that they can improve and change social and environmental problems, they will actively engage in green food consumption behavior. Thus, business managers should plan and implement marketing strategies to integrate green food consumption with consumers’ perceptions of effectiveness. For example, marketing advertising may trigger consumers’ perceptions of environmental protection and combine this with their green food purchasing behavior (e.g., combining organic food consumption with environmental protection and bridging green food purchases with reducing pollution). Furthermore, policymakers should launch different strategies to persuade consumers to engage in green food consumption. For example, government agencies may use advertising to persuade consumers that if they purchase environmentally friendly food products, then they will contribute greatly to the improvement of the environment and society. In addition, policymakers should plan different strategies to enhance consumers’ trust in society because when consumers trust their society, they will engage in positive activities that benefit the entire society. The findings of this study also provide implications for individual consumers. Individuals should trust our society and believe that each consumer can contribute to our society in solving environmental and social issues. We can experience a happy life and create value for our entire society by engaging in green food consumption.

## 6. Conclusions

To the best of the researchers’ knowledge, this study is the first attempt to examine a mediating and moderating mechanism in the relationship between perceived consumer effectiveness and psychological wellbeing. Empirical results reveal several fresh insights. Perceived consumer effectiveness has a positive impact on psychological wellbeing. Green food consumption appears to have a mediating effect on the relationship between perceived consumer effectiveness and psychological wellbeing. Furthermore, social trust has a moderating effect on the relationship between perceived consumer effectiveness and green food consumption. Notably, social trust also moderates the indirect effect of perceived consumer effectiveness on psychological wellbeing through green food consumption.

Some limitations should be acknowledged and overcome in future research. For example, cross-sectional data may affect the causal relationship among variables in this study. Hence, future research should collect longitudinal data to validate such a relationship to overcome this limitation. Furthermore, cross-sectional data may generate a common method bias that influences the results of the hypothesis testing. We suggest that future research should obtain consumers’ contact and collect data from them several times. In addition, this study analyzed data from consumers in China only, which may limit the generalizability of the results. Thus, future research should collect data from consumers in different emerging markets (e.g., Southeast Asia, Russia, and Brazil).

## Figures and Tables

**Table 1:** Items and constructs of measures.

Construct	Items	Source
Perceived consumer effectiveness	What I purchase as a consumer affects the nation’s environmental problem. Each consumer’s behavior can affect how companies treat their employees. Each consumer can have a positive effect on society by purchasing products sold by socially responsible companies. Given that one person cannot have any effect on pollution and natural resource problems, what I do doesn’t make any difference (r).	[48]
Green food consumption	I always buy green food. I always try to buy food with green labels. I buy green food even at higher prices. I recommend green food that I have consumed to my relatives and friends.	[4]
Social trust	In the last month, how often did you feel that the way our society works makes sense to you. In the last month, how often did you feel that our society is becoming a better place.	[16]
Psychological wellbeing	In most ways, my social life is close to my ideal. I am satisfied with my social life. So far, I have the important things that I want in my social life. If I could live my social life again, then I would change almost nothing.	[49]

**Table 2:** Sample characteristics (*N* = 514).

Variable	Frequency	Percentage
Gender		
Male	177	34.4%
Female	337	65.6%
Age		
Under 20	102	19.8%
20–under 30	298	58.0%
30–under 40	66	12.8%
41 and above	48	9.3%
Marital status		
Married	150	29.2%
Not married	362	70.4%
Education		
High school and below	153	29.8%
University	243	66.7%
Master and above	18	3.5%
Income		
Under 500 USD	124	24.1%
500–under 1000 USD	348	67.7%
1000 USD or above	42	8.2%

**Table 3:** Means, standard deviation, and Pearson correlation (*N* = 514).

Variable	Means	SD	1	2	3	4
Perceived consumer effectiveness	3.86	0.71	0.76			
Green food consumption	3.74	0.74	0.53 **	0.78		
Social trust	3.69	0.83	0.48 **	0.51 **	0.85	
Psychological wellbeing	3.59	0.78	0.41 **	0.27 **	0.47 **	0.74

** *p* <0.01

**Table 4:** Results of the measurement model (*N* = 514).

Variable	Items	Factor Loadings	CR Value	AVE Value	Cronbach’s α
Perceived consumer effectiveness (PCE)	PCE1	0.65 ***	0.84	0.57	0.84
	PCE2	0.80 ***			
	PCE3	0.77 ***			
	PCE4	0.80 ***			
Green food consumption (GFC)	GFC1	0.81 ***	0.86	0.61	0.90
	GFC2	0.79 ***			
	GFC3	0.81 ***			
	GFC4	0.72 ***			
Social trust (STR)	STR1	0.85 ***	0.84	0.72	0.84
	STR2	0.85 ***			
Psychological wellbeing (PSW)	PSW1	0.62 ***	0.83	0.55	0.77
	PSW2	0.63 ***			
	PSW3	0.83 ***			
	PSW4	0.85 ***			

*** *p* <0.001.

**Table 5:** Moderating role of social trust (*N* = 514).

Moderator	Perceived Consumer Effectiveness (X) → Green Food Consumption (M) → Psychological Wellbeing (Y)				
	Stage		Effect		
	First (P_XM_)	Second (P_MY_)	Direct Effects (P_XY_)	Indirect Effects (P_XM_P_MY_)	Total Effects (P_XY_ + P_XM_P_MY_)
Low social trust	0.557 ***	0.391 ***	0.062 *	0.218 ***	0.280 **
High social trust	0.629 ***	0.438 **	0.098 *	0.276 ***	0.374 **
Differences	0.072 ***	0.047 ***	0.036	0.058 **	0.094 **

* *p* < 0.05, ** *p* < 0.01, *** *p* < 0.001; P_XM_: path from independent variable to mediator; P_MY_: path from mediator to dependent variable; P_XY_: path from independent variable to dependent variable.
